# Comparison of diagnostic accuracy among procalcitonin, C-reactive protein, and interleukin 6 for blood culture positivity in general ICU patients

**DOI:** 10.1186/s13054-018-2269-5

**Published:** 2018-12-17

**Authors:** Qin Wu, Hao Yang, Yan Kang

**Affiliations:** Department of Critical Care Medicine, West China Hospital, Sichuan University, Chengdu, China

Dear editor,

Despite various technological advances, it still usually takes at least 24 to 48 h to obtain a blood culture result. The subsequent delays in diagnosis and treatment of infection can negatively impact care in the intensive care unit (ICU). Biomarkers, such as procalcitonin (PCT), have been suggested as predictors of blood culture positivity in patients with different diseases [1, 2]. However, the performances of PCT, C-reactive protein (CRP), and interleukin 6 (IL-6) in the prediction of blood culture positivity have never been assessed in a general ICU population.

We retrospectively assessed 13,377 consecutive adult ICU patients who did not have chronic kidney disease and who were discharged from a Chinese teaching hospital between January 1, 2016 and December 31, 2017. Among them, 534 patients who had suspected infection and whose PCT, CRP, and IL-6 levels were measured when their blood was drawn for culture in accordance with standard procedure were analyzed. After the false-positive culture results as judged by two independent clinicians were excluded, the rate of positive blood culture was 7.49% (40 out of 534). Baseline characteristics between the blood culture–positive and blood culture–negative groups were comparable (Additional file 1). In contrast, the levels of PCT and IL-6 in the blood culture–positive group were significantly higher than those in the blood culture–negative group (Fig. 1). Receiver operating characteristic curves in Additional file 1 showed areas under the curve of 0.593 (95% confidence interval (CI) 0.494–0.692) for PCT, 0.520 (95% CI 0.400–0.693) for CRP, and 0.537 (95% CI 0.457–0.617) for IL-6. A logistic regression analysis revealed that levels of PCT of more than 2.435 ng/mL and IL-6 of more than 264.95 ng/mL were independently associated with blood culture positivity. Moreover, when those two biomarkers were combined, values of PCT of more than 2.435 ng/mL and IL-6 of more than 264.95 ng/mL could result in a very high specificity (93.57%) but low sensitivity (12.5%).

**Fig. 1:**
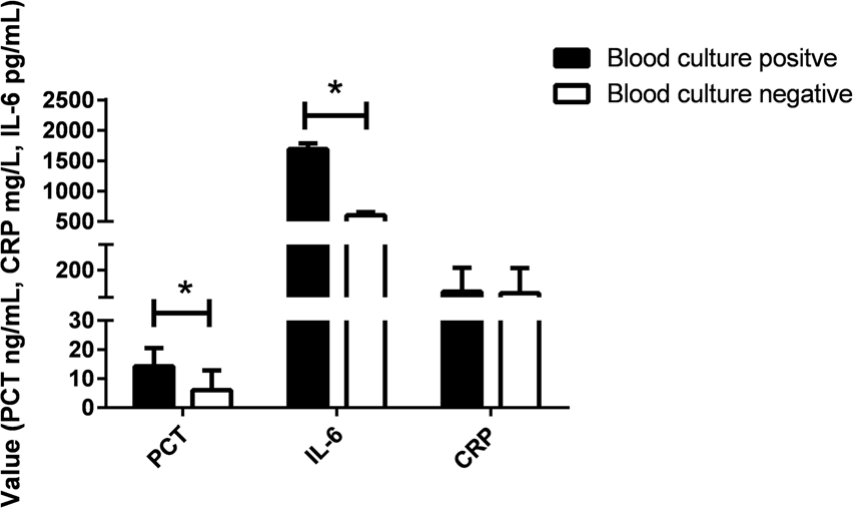
Plot of procalcitonin (PCT), C-reactive protein (CRP), and interleukin 6 (IL-6) plasma concentrations in blood culture–positive and blood culture–negative groups. Significant differences (indicating as asterisk) in PCT and IL-6 were observed between groups

Our results suggest that patients with suspected infection probably have positive blood culture results if both their PCT and IL-6 levels are high. Even with the limitation of a single-center experience, our results indicate that PCT, IL-6, and PCT plus IL-6 levels might be useful for helping physicians to rapidly identify patients who are at risk of bloodstream infection and to select an appropriate empirical therapy.

## Additional file

Additional file 1: **Table S1.** Demographics, clinical, and outcome data of patient cohort. (DOCX 30 kb)

## Abbreviations

CI: Confidence interval
CRP: C-reactive protein
ICU: Intensive care unit
IL-6: Interleukin 6
PCT: Procalcitonin
