# Supplementary material for: Comparison of diagnostic accuracy among procalcitonin, C-reactive protein, and interleukin 6 for blood culture positivity in general ICU patients
Source: Crit Care. 2018 Dec 17;22:339. doi: 10.1186/s13054-018-2269-5 (PMC6296063; doi:10.1186/s13054-018-2269-5)
Supplement: Supplementary file 1 — Table S1. Demographics, clinical, and outcome data of patient cohort. (DOCX 30 kb) [file 13054_2018_2269_MOESM1_ESM.docx]

**Table S1 Demographics, Clinical and Outcome data of patient cohort**

| **Parameters** | **All**  **(n=534)** | **Blood culture negative**  **(n=494)** | **Blood culture positive**  **(n=40)** | **P** |
| --- | --- | --- | --- | --- |
| Sociodemographic |  |  |  |  |
| Age, year (mean±SD) | 58.08 (16.79) | 58.78 (16.43) | 57.45 (17.44) | 0.809 |
| Male, n (%) | 319 (59.74) | 294 (59.51) | 25 (52.50) | 0.711 |
| Married, n (%) | 413 (77.34) | 384 (77.73) | 29 (72.50) | 0.447 |
| Clinical features at admission |  |  |  |  |
| APACHEⅡScore | 21.95 (10.44) | 21.50 (9.52) | 22.10 (13.82) | 0.164 |
| Outcome |  |  |  |  |
| Hospital LOS, day (mean±SD) | 22.03 (26.73) | 21.14 (29.98) | 23.09 (21.69) | 0.121 |
| ICU LOS, day (mean±SD) | 17.24 (13.82) | 17.03 (11.46) | 17.98 (16.73) | 0.265 |
| In-hospital mortality, n(%) |  |  |  | <0.001 |
| Live | 315 (58.98) | 300 (60.70) | 15 (37.50) |  |
| Dead | 63 (11.79) | 48 (9.70) | 15 (37.50) |  |
| Discharge against order | 156 (29.23) | 146 (29.60) | 10 (25.00) |  |
| Ventilation-free day, day (mean±SD) | 20.51 (5.68) | 20.56 (6.77) | 20.43 (6.45) | 0.891 |
| Hospital costs, CHY (mean±SD) | 131,283 (89,205) | 127,372 (99,269) | 138,371 (98,592) | 0.026 |
| Complete blood count* |  |  |  |  |
| RBC, mean (SD),×10^9^/L | 3.50 (0.78) | 3.50 (0.78) | 3.50 (0.73) | 0.123 |
| HGB, mean (SD), g/L | 102.66 (23.27) | 103.12 (23.13) | 96.98 (24.55) | 0.108 |
| HCT, mean (SD), L/L | 0.32 (0.69) | 0.32 (0.69) | 0.30 (0.68) | 0.128 |
| MCV, mean (SD), fL | 91.02 (7.18) | 91.03 (7.24) | 90.96 (6.46) | 0.954 |
| MCH, mean (SD), pg | 29.58 (2.57) | 29.60 (2.54) | 29.30 (2.41) | 0.474 |
| PLT, mean (SD),×10^9^/L | 146.57 (92.97) | 144.86 (91.36) | 167.63 (110.41) | 0.136 |
| WBC, mean (SD),×10^9^/L | 12.02 (7.32) | 11.91 (7.24) | 13.39 (8.12) | 0.218 |
| Coagulation test* |  |  |  |  |
| INR, mean (SD) | 1.37 (0.73) | 1.37 (0.75) | 1.36 (0.48) | 0.941 |
| PT, mean (SD), s | 16.08 (8.23) | 16.08 (8.43) | 16.00 (5.55) | 0.953 |
| APTT, mean (SD), s | 43.20 (20.22) | 43.05 (19.92) | 44.68 (23.38) | 0.631 |
| Fib, mean (SD), g/L | 3.69 (1.87) | 3.72 (1.86) | 3.37 (1.91) | 0.255 |
| TT, mean (SD), s | 19.37 (8.29) | 19.46 (8.62) | 18.47 (3.38) | 0.483 |
| Arterial Blood Gas Test* |  |  |  |  |
| pH, mean (SD) | 7.20 (0.57) | 7.20 (0.57) | 7.23 (0.53) | 0.756 |
| PaO2, mean (SD), mmHg | 99.97 (40.49) | 99.78 (40.84) | 101.95 (37.00) | 0.746 |
| PaCO2, mean (SD), mmHg | 39.74 (10.02) | 39.89 (9.96) | 38.20 (10.72) | 0.308 |
| BE, mean (SD), mmol/L | -0.47 (5.29) | -0.37 (5.34) | -1.59 (4.61) | 0.164 |
| Lac, mean (SD), mmol/L | 2.89 (2.59) | 2.92 (2.67) | 2.59 (1.74) | 0.450 |
| Biochemical analysis* |  |  |  |  |
| Serum sodium, mean (SD), mmol/L | 140.64 (7.62) | 140.62 (7.52) | 140.91 (8.74) | 0.816 |
| Serum potassium, mean (SD), mmol/L | 4.01 (0.64) | 4.01 (0.65) | 3.99 (0.49) | 0.825 |
| Serum chloride, mean (SD), mmol/L | 107.66 (7.79) | 107.62 (7.74) | 108.07 (8.45) | 0.732 |
| TB, mean (SD), mmol/L | 22.04 (28.28) | 21.46 (27.59) | 29.16 (35.38) | 0.098 |
| DB, mean (SD), mmol/L | 14.55 (25.38) | 14.05 (24.87) | 20.74 (30.67) | 0.109 |
| ALP, mean (SD), mmol/L | 85.22 (78.04) | 91.96 (66.31) | 125.43 (160.68) | 0.097 |
| TP, mean (SD), mmol/L | 52.43 (9.27) | 52.42 (9.21) | 52.52 (10.02) | 0.951 |
| Creatinine, mean (SD), mmol/L | 103.43 (97.23) | 104.22 (98.51) | 93.70 (80.27) | 0.511 |
| Cys-c, mean (SD), mmol/L | 1.24 (0.74) | 1.25 (0.77) | 1.16 (0.76) | 0.497 |
| GGT, mean (SD), mmol/L | 66.58 (98.70) | 65.34 (99.65) | 81.83 (85.85) | 0.310 |

*Measured when drawing blood for culture

RBC: Red blood cell, HGB: Hemoglobin, HCT: Hematocrit, MCV: Mean corpuscular volume, MCH: Mean corpuscular hemoglobin, MCHC: Mean corpuscular hemoglobin concentration, PLT: Platelets, WBC: white blood cell; INR: International normalized ratio, PT: prothrombin time, APTT: Activated partial thromboplastin time, Fib: fibrinogen, TT: Thrombin time, PaO2: Arterial oxygen partial pressure, PaCO2: Arterial carbon dioxide partial pressure, BE: Base excess, Lac: lactate, TB: Total bilirubin, DB: Direct bilirubin, IB: Indirect bilirubin, ALP: Alkaline Phosphatase, TP: Total protein, Cys-c: Cystatin C, GGT: Gamma-Glutamyl Transferase,
